# Acute spontaneous neck haematoma in children: a rare entity

**DOI:** 10.1186/s12887-015-0356-1

**Published:** 2015-04-11

**Authors:** Shimin Zhuang, Jin Ye, Jingjia Li

**Affiliations:** Department of Thyroid-Head & Neck Surgery, The Second Affiliated Hospital to Nan-Chang University, 1 Min De Road, Nan Chang, Jiang Xi Province 330000 People’s Republic of China; Department of Otorhinolaryngology Head and Neck Surgery, the Third Affiliated Hospital of Sun Yat-sun University, Guangzhou, 510630, Guangdong Peoples Republic of China

**Keywords:** Spontaneous haematoma, Neck, Children

## Abstract

**Background:**

Acute spontaneous neck haematoma is rare in children. This rare type of hematoma occurs abruptly without any preceding trauma or iatrogenic damage, making it very difficult to determine the cause precisely. We report here the first two cases of acute spontaneous neck haematoma presenting with neck swelling, and discuss in this article the diagnosis and treatment strategy in our patients.

**Case presentation:**

We report a 19-month-old girl and a 30 month-old boy with neck swelling for 10 days. There was no history of trauma, cough, excessive muscular strain or iatrogenic injury, and both patients were not on any anticoagulants or antiplatelet drugs. On initial examination, the swelling was tender, firm and not mobile on palpation. A definite diagnosis was made by hematoma puncture. Both of the haematoma resolved spontaneously in two weeks without any complications or sequelae.

**Conclusions:**

Acute spontaneous neck hematoma in children is a rare disorder without any etiology or precipitating factors. The difficulty in making an early diagnosis is mainly due to the nonspecific presenting symptoms. Conservative management and follow-up is recommended as a choice of treatment.

## Background

Acute spontaneous neck haematoma is rare in children. This rare type of hematoma occurs abruptly without any preceding trauma or iatrogenic damage. Presenting symptoms are usually nonspecific, making it difficult to get a definite diagnosis. A MEDLIINE literature search did not reveal any reported cases of spontaneous haematoma in the neck without any precipitating factor in children. We report herein two cases of spontaneous neck haematoma in a 19-month-old girl and a 30-month-old boy who initially presented with neck swelling.

## Case presentation

**Figure 1:**
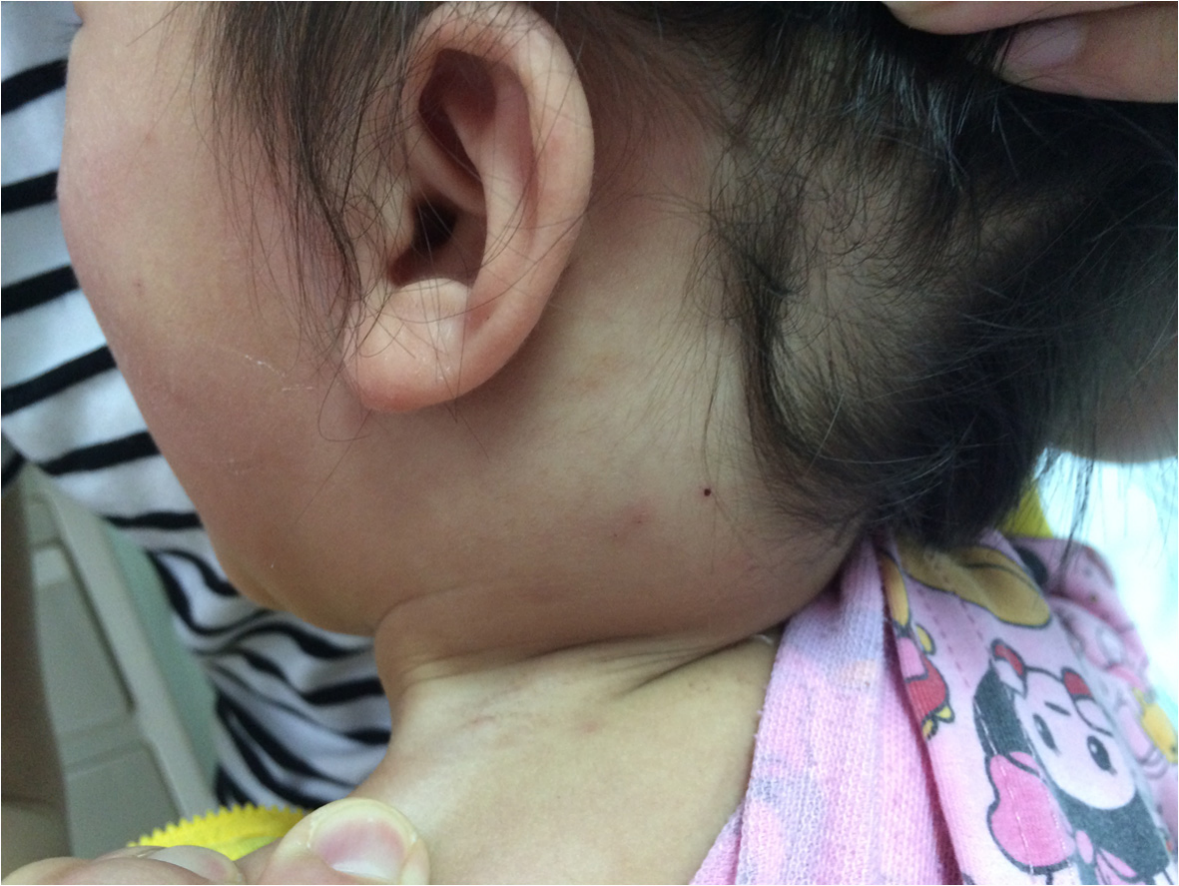
A 4.5 × 3.5 cm neck mass was seen in the posterior triangle of the left side, the skin over the swelling was normal.

Case 1. A 19-month-old girl presented with post left-sided neck swelling, fever and pain for 7 days. She started with fever at home and the swelling appeared spontaneously after 24 h, and gradual increase in 4 days. There was no history of trauma, cough, excessive muscular strain or iatrogenic injury. She was not on any anticoagulants or antiplatelet drugs. The general examination was normal. A 4.5 × 3.5 cm spherical swelling was seen on the trapezius in the posterior triangle of the left side of the neck and the skin over the swelling was normal (Figure 1). There was no local rise of temperature but the swelling was mildly tender, firm, not pulsatile and not mobile on palpation. The neck and shoulder movements were normal and no peripheral neurological deficit was present.

Case 2. A 30-month-old boy was transferred to our institution due to up left-sided neck swelling for 10 days. Presenting symptoms like case 1, presented with a rapidly neck swelling and enlarging but without fever. Past history did not reveal any trauma to the head, nor any bleeding disorder or pertinent family history. He was not on any anticoagulants or antiplatelet drugs. The patient was afebrile, and the neurological examination showed normal. A 5 × 4 cm spherical swelling was seen on up left-sided neck.

The laboratory tests indicated high inflammatory reactions in case 1. The haematological and biochemical investigations were normal in case 2. All the patients coagulation function and bleeding times were both normal. Taken together, acute bleeding of the neck was suspected.

**Figure 2:**
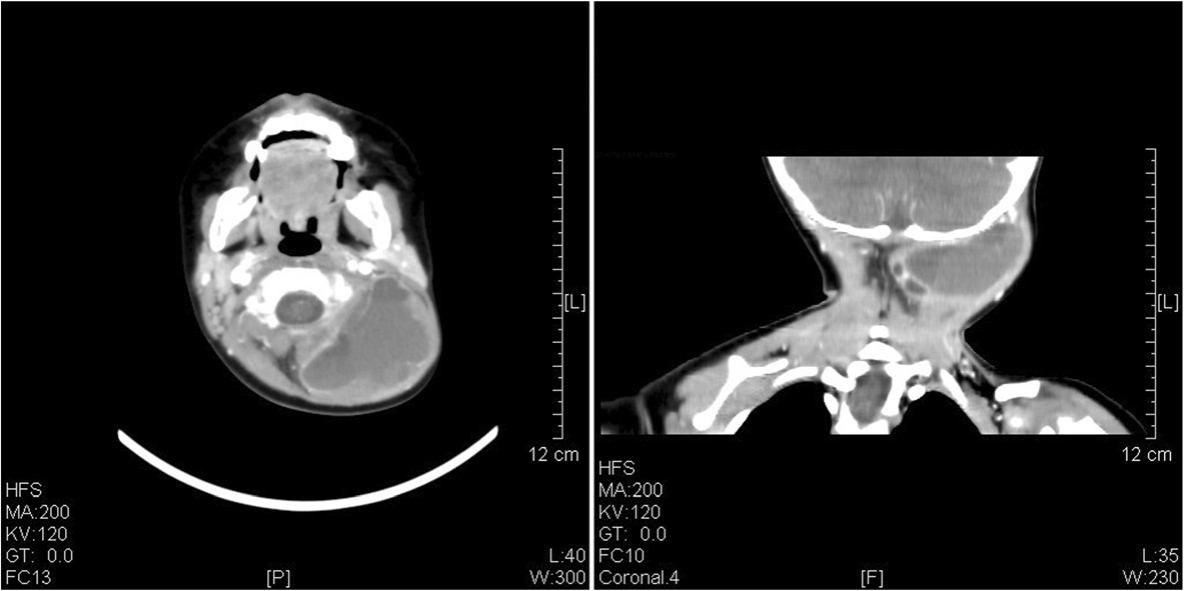
A contrast CT scan revealed a low-density lesion without enhancement in the left occipitalia in case 1.

**Figure 3:**
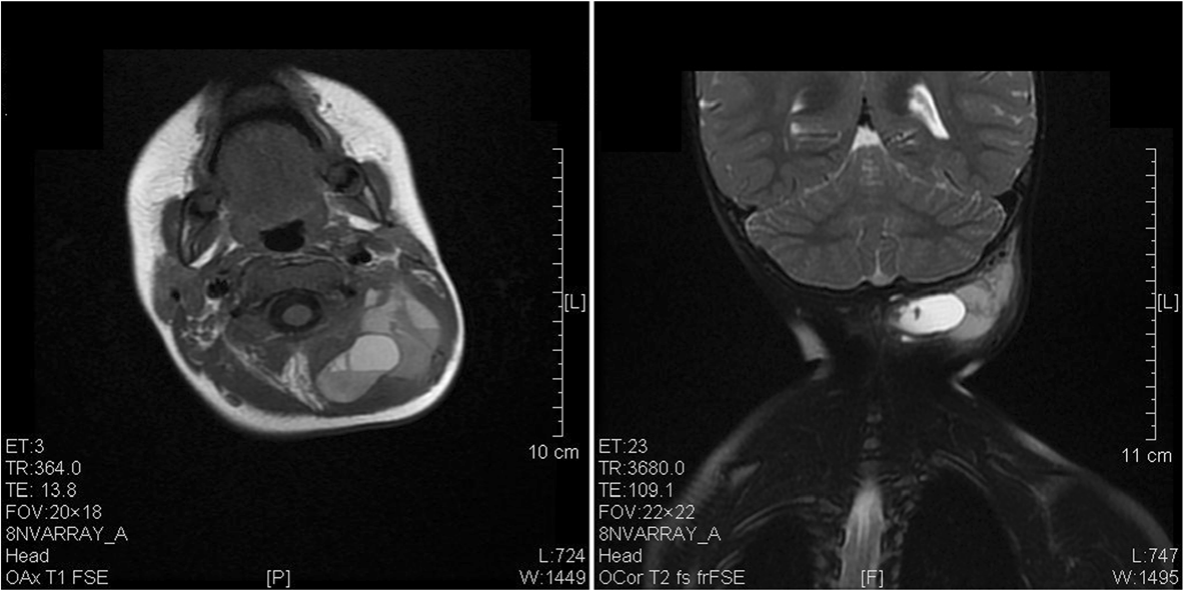
A magnetic resonance image showed an enlarged heterogenous mass in the left occipitalia in case 1.

A computed tomography (CT) scan with contrast of the neck was reported as resolving haematoma 4.3 cm deep to the left occipitalia at the level of lobulus auriculae in case 1 (Figure 2), and magnetic resonance imaging (MRI) of the head with contrast demonstrated a posterior, lobulated mass extending to the left occipitalia (Figure 3). All the CT and MRI did not show any evidence of destructive change or fracture of the vertebral body and neural arch.

**Figure 4:**
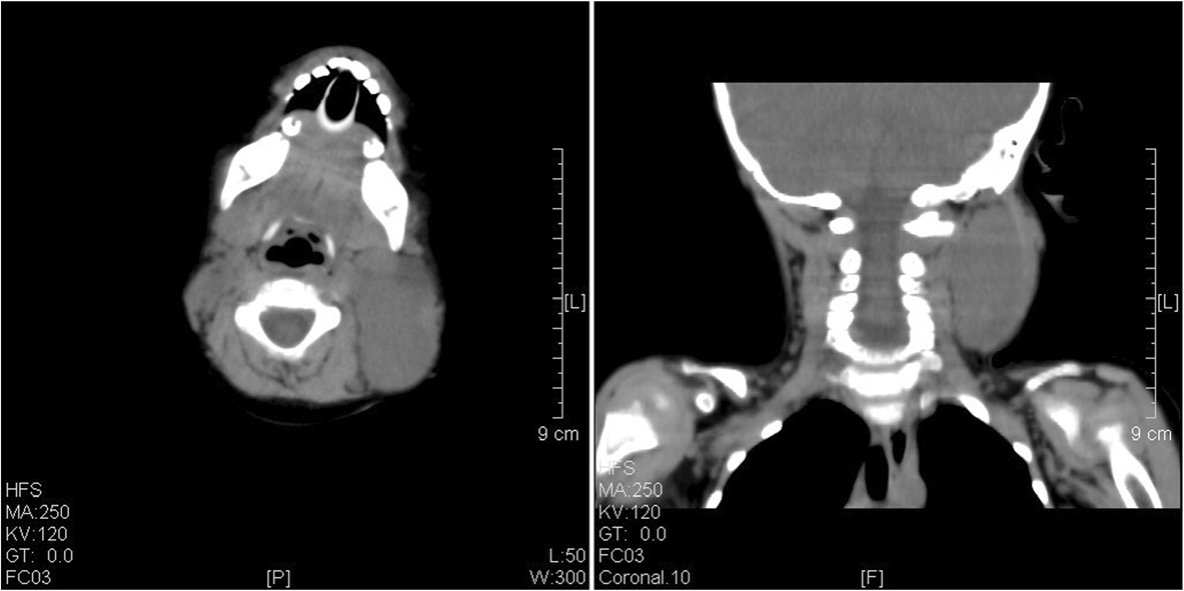
A CT scan showed a haematoma under the sternocleidomastoid muscle in case 2.

A CT scan of the neck was reported as resolving haematoma 4.9 cm deep to the left sternocleidomastoid at the level of above the hyoid bone in case 2 (Figure 4). The CT scan did not show any evidence of destructive change or fracture of the vertebral body and neural arch.

A definite diagnosis of acute spontaneous neck haematoma was made by hematoma puncture in the two children. During this procedure, the aspiration was performed with 5 ml syringe, and the diagnosis was achieved by the successful aspiration of bleeding liquid.

In case 1, the patient was treated with analgesics. No hemostatic was used. Both patient received treatment of fine needle aspiration of bleeding liquid form, but the hematoma expanded rapidly in a few hours. Thus, we decided to wait for a time for spontaneous resolution. The lesion resolved spontaneously two weeks later, without any complications or sequelae.

## Discussion

It is rare to see spontaneous haematoma in the neck without any comorbidity. To the best of our knowledge, this is the first report of acute spontaneous neck haematoma presenting as swelling in children. In reported cases, haematoma was associated with trauma, bleeding diathesis, invasive procedure or surgery [1,2]. They commonly occurred in the anterior triangle of the neck, causing airway compromise or dysphagia. Spontaneous cervical epidural haematoma is a well-known neurosurgical emergency. Although this condition occurs in all ages, it has preponderance in older aged patients who have received anticoagulants or antiplatelet drugs and therefore have bleeding or coagulation defect tendencies. While the lesion is located in the thoracolumbar lesion in older patients, the cervicothoracic site is more common in children [3]. In our report, two babies had haematoma in the neck.

Variety of intrinsic factors such as prolonged coughing, sneezing and vomiting are implicated in the cause [1]. The neck spaces communicate with each other and this allows spread of collection from the skull base to the mediastinum [2]. The airway obstruction is potentially possible if there is spread of collection into the anterior triangle of the neck or mediastinum. The most common cervical haematomas in patients who were undergoing anticoagulation therapy are laryngeal, retropharyngeal and sublingual [4]. Paradoxically, the patient’s coagulation function and bleeding times were both normal in the literature, and the haematological and biochemical investigations were normal. Taken together, acute spontaneous neck haematoma was diagnosed in children with no etiology.

MRI and CT with contrast are the choice of imaging modality and plays an important role in diagnosis and evaluation of spontaneous haematoma [5,6]. Imaging scan not only can delineate the site of the haematoma, but will identify the source of the haematoma. The signal intensity difference in the MRI allows estimation of the stage of hematoma. In our case, the hematoma was seen as a posterior slight low signal intensity lesion on the T1 weighted image, and on the T2 weighted image as a high signal intensity lesion suggesting a subacute type hemorrhage of more than 3 days progression, and thus coincides with the a few days duration of symptom onset. CT scan also can indicate the presence of a low-density lesion extending from the neck swelling. No gas bubbles were observed in the lesion.

No standardized treatment and follow-up is established for patients with acute spontaneous neck hematomas in children. No recurrent cases have been reported. In our document the patients after evaluation of radiologic examinations, no certain diagnosis could be made; we thought that the patient might be experiencing spontaneous neck hematomas with unknown etiology. The patients herein were treated with analgesics and diagnostic fine needle tap bleeding liquid form haematoma. But the size of the lesion had enlarged day by day as the surgery before. Despite no experience in acute spontaneous neck hematomas in children, we decided to wait for a time for spontaneous resolution because excision of the lesion might result in permanent surgical scars in the neck. As expected, the lesion resolved spontaneously in two weeks by natural absorption, without any complications or sequelae.

## Conclusions

Acute spontaneous neck hematoma in children is a rare disorder without any etiology or precipitating factors. The difficulty in making an early diagnosis is mainly due to the nonspecific presenting symptoms. In such conditions, MRI or CT imaging scan should be considered to make a differential diagnosis. Conservative management and follow-up is recommended as a choice of treatment.

## Consent

Written informed consent was obtained from the patient for publication of this Case report and any accompanying images. A copy of the written consent is available for review by the Editor of this journal.

## Abbreviations

CT: Computed tomography
MRI: Magnetic resonance imaging

## References

[1] Schroder KE, Mair WS (1978). Spontaneous haematoma in the head and neck. J Laryngol Otol.

[2] Al-fallouji HK, Snow DG, Kuo MJ, Johnson PJ (1993). Spontaneous retropharyngeal haematoma: two cases and a review of the literature. J Laryngol Otol.

[3] Patel H, Boaz JC, Phillips JP, Garq BP (1998). Spontaneous spinal epidural hematoma in children. Pediatr Neurol.

[4] Difrancesco RC, Escamilla JS, Sennes LU, Voegles RL, Tsuji DH (1999). Spontaneous cervical hematoma: a report of two cases. Ear Nose Throat J.

[5] Crisi G, Sorgato P, Colombo A, Scarpa M, Falasca A, Anqiari P (1990). Gadolinium-DTPA-enhanced MR imaging in the diagnosis of spinal epidural hematoma. Report of a case Neuroradiology.

[6] Pai SB, Maiya PP (2006). Spontaneous spinal epidural hematoma in a toddler-a case report. Childs Nerv Syst.

